# The glymphatic system's role in linking cardiometabolic health to cerebral small vessel disease and frailty in Chilean healthy older adults

**DOI:** 10.1002/alz70857_106737

**Published:** 2025-12-26

**Authors:** Ana Maria Castro Laguardia, Daniel Franco O'Byrne, Cecilia Gonzalez Campo, Paul H Delano, Gonzalo Farías, Agustin Ibanez, Carolina Delgado, Vicente Medel

**Affiliations:** ^1^ Latin American Brain Health Institute (BrainLat), Universidad Adolfo Ibáñez, Santiago, Región Metropolitana de Santiago, Chile; ^2^ Center for Social and Cognitive Neuroscience, Adolfo Ibañez University, Santiago, Región Metropolitana, Chile; ^3^ Latin American Brain Health Institute (BrainLat), Universidad Adolfo Ibáñez, Santiago, Región Metropolitana, Chile; ^4^ CONICET, Buenos Aires, Argentina; ^5^ Cognitive Neuroscience Center (CNC), Universidad de San Andrés, Buenos Aires, Buenos Aires, Argentina; ^6^ Universidad de Chile, Santiago, Chile; ^7^ University of Chile, Santiago, Chile; ^8^ Global Brain Health Institute (GBHI), Trinity College Dublin (TCD), Dublin, Dublin, Ireland; ^9^ Latin American Brain Health Institute (BrainLat), Universidad Adolfo Ibañez, Santiago, Chile; ^10^ Global Brain Health Institute (GBHI), University of California San Francisco (UCSF); & Trinity College Dublin, Dublin, Ireland; ^11^ Universidad de Chile, Santiago, Santiago, Chile; ^12^ Hospital Clínico Universidad de Chile, Departamento de Neurología y Neurocirugía, Santiago, Santiago, Chile

## Abstract

**Background:**

Cardiometabolic factors such as elevated blood pressure, body mass index, cholesterol, and glucose levels are well‐known for their role in cardiovascular disease risk, but they also significantly impact brain structure and function, particularly in aging populations. These factors contribute to cerebral small vessel disease (cSVD), a leading cause of vascular cognitive impairment and dementia. Key neuroimaging markers of cSVD include white matter hyperintensities (WMH) and enlarged perivascular spaces (PVS), which are increasingly studied due to their role in the glymphatic system, responsible for brain waste clearance. Enlarged PVS are believed to signal glymphatic dysfunction, contributing to the accumulation of metabolic waste and cSVD development. Moreover, cardiometabolic health is linked to frailty, often assessed through gait speed (GS), with slower GS indicating higher frailty and cardiometabolic risk, reflecting a complex relationship between brain health, physical frailty, and cardiometabolic factors. Despite these findings, the mechanisms connecting cardiometabolic burden, cSVD, and frailty remain unclear. This study aims to explore the relationship between a cardiometabolic index, cSVD markers (PVS and WMH), and GS in healthy older adults from Chile.

**Method:**

The sample comprised 87 healthy Chilean older adults (mean age 73.5 years, 58 women). The cardiometabolic index was derived from blood pressure, body mass index, cholesterol, and glucose levels. PVS volume was calculated using an adaptation of an automated method by Sepehrband et al. (2019), while WMH was measured in FLAIR images using the lesion segmentation tool (LST) on SPM12. Spearman correlations were performed between variables, and mediation analyses were conducted for two models: PVS‐cardiometabolic index (mediator) and GS, and WMH‐cardiometabolic index (mediator) and GS.

**Results:**

There was no significant correlation between PVS and WMH. However, the mediation analysis involving PVS, the cardiometabolic index, and GS revealed significant direct and indirect effects of the cardiometabolic index on the relationship between PVS and GS. In contrast, the analysis for WMH did not show significant results, indicating no mediating effect of the cardiometabolic index in this relationship.

**Conclusion:**

This research underscores the complex links between cardiometabolic health, glymphatic dysfunction (with PVS as a marker), and frailty, highlighting the differential contributions of cSVD markers to physical frailty in older adults.

